# Global collaboration and innovation in malignant cerebral edema research: a bibliometric perspective

**DOI:** 10.3389/fneur.2025.1624101

**Published:** 2025-07-23

**Authors:** Xinhua Peng, Rongrong Zhu, Ke Zhang, Jianghong Ji, Chuanguo Lv, Feng Feng

**Affiliations:** ^1^Medical School of Nantong University, Nantong, China; ^2^Imaging Department, Qidong People’s Hospital, Qidong Liver Cancer Institute, Affiliated Qidong Hospital of Nantong University, Qidong, China; ^3^Department of Radiology, Affiliated Tumor Hospital of Nantong University, Nantong, China

**Keywords:** malignant cerebral edema, decompressive craniectomy, cytotoxic edema, blood–brain barrier, neurocritical care, bibliometric analysis, precision medicine, global collaboration

## Abstract

**Aim:**

Malignant cerebral edema (MCE) is a life-threatening complication of acute brain injuries, with mortality rates exceeding 80% in the absence of treatment. Despite advancements in osmotic therapies and decompressive craniectomy (DC), MCE continues to pose substantial clinical challenges. This study systematically maps the evolution of MCE research (2005–2024) to identify key trends, research gaps, and future priorities.

**Methods:**

A bibliometric analysis of 1,460 peer-reviewed articles from the Web of Science Core Collection was conducted using CiteSpace, VOSviewer, and Bibliometrix. Key metrics included publication trends, geographic and institutional contributions, keyword co-occurrence, citation networks, and co-authorship patterns.

**Results:**

Annual publications increased from 55 in 2005 to 128 in 2024, progressing through three distinct phases: Foundational growth (2005–2009), consolidation (2010–2014), and rapid expansion (2015–2024). The United States (28.9%) and China (18.7%) dominated research output, with Harvard University and the University of California System leading institutional collaboration clusters. High-impact journals highlighted clinical advancements, including Stroke (h-index = 27). Keyword analysis demonstrated a thematic progression from blood–brain barrier pathophysiology to clinical innovations, including DC and emerging predictive modeling techniques incorporating machine learning. Landmark trials, including DECIMAL and HAMLET, validated early surgical intervention, while emerging trends have emphasized precision medicine and artificial intelligence (AI)-driven risk stratification.

**Conclusion:**

The MCE research has transitioned from foundational pathophysiology to interdisciplinary clinical practice and data integration. However, critical gaps remain, including underrepresentation in pediatric research, disparities in global neurocritical care, and challenges in translational application. Future priorities should focus on biomarker discovery, equitable global collaborations, and AI-enhanced frameworks to transform survival into functional recovery worldwide.

## Introduction

Malignant cerebral edema (MCE) is a severe complication that occurs in the context of acute brain injuries—most notably large hemispheric infarctions—and is characterized by rapid and progressive brain swelling (1). Without timely intervention, this pathological swelling can lead to cerebral herniation, coma, and death (2, 3). The reported incidence of MCE following massive ischemic strokes ranges from 10 to 30%, with untreated cases exhibiting mortality rates exceeding 80% (4, 5). MCE pathophysiology involves cytotoxic edema caused by cellular energy failure, ionic pump dysfunction, and vasogenic edema resulting from blood–brain barrier (BBB) breakdown and endothelial injury. Recent evidence has also emphasized the role of astrocyte-regulated water transport through aquaporin-4 (AQP4) channels, linking neuroinflammation and glial activation to worsening edema (6, 7). Despite the application of osmotic agents (mannitol, hypertonic saline) and decompressive craniectomy (DC) as mainstay treatments, these interventions remain limited by variable efficacy, delayed implementation, and significant long-term morbidity (8, 9). The absence of reliable early biomarkers and the inability to stratify patients at risk for malignant progression further complicate clinical decision-making.

Over the past two decades, parallel advancements have been made in both clinical and basic research domains. Clinically, predictive models incorporating ASPECTS scores and midline shift metrics have been developed, and landmark trials (DECIMAL, DESTINY II) have demonstrated survival benefits of hemicraniectomy in selected populations (10–13). Concurrently, preclinical studies identified novel therapeutic targets, including SUR1-TRPM4 channel inhibition (glibenclamide) and dynamic neuromonitoring of the neurovascular units (14, 15). However, significant translational gaps persist between these domains: Clinical guidelines often rely on single-center observational data, while mechanistic discoveries lack robust translational validation platforms. This disconnect is further compounded by two fundamental challenges: the lack of validated early biomarkers for malignant transformation and striking geographic disparities in research output, with over 70% of studies originating from high-income countries. A systematic analysis of knowledge evolution patterns and collaborative networks could yield strategic insights for optimizing global neurocritical care.

We conducted the first bibliometric analysis of MCE research to address this knowledge gap, analyzing 1,460 peer-reviewed publications indexed in the Web of Science Core Collection (WOSCC; 2005–2024). Using temporal trend analysis, country- and institution-level mapping, and keyword co-occurrence network construction, our objectives were as follows: (1) delineating the major intellectual structures and emerging research clusters—including glibenclamide-based therapies, neuromonitoring technologies, and neuroinflammation; (2) visualizing international and interdisciplinary collaboration patterns; (3) outlining a forward-looking research agenda prioritizing precision medicine, biomarker discovery, and equitable access to neurocritical care. By synthesizing fragmented literature and mapping the trajectory of MCE research, this study provides actionable insights for clinicians, researchers, and policymakers seeking to reduce the global burden of this often fatal complication.

## Methods

### Data source and search strategy

**Figure 1:**
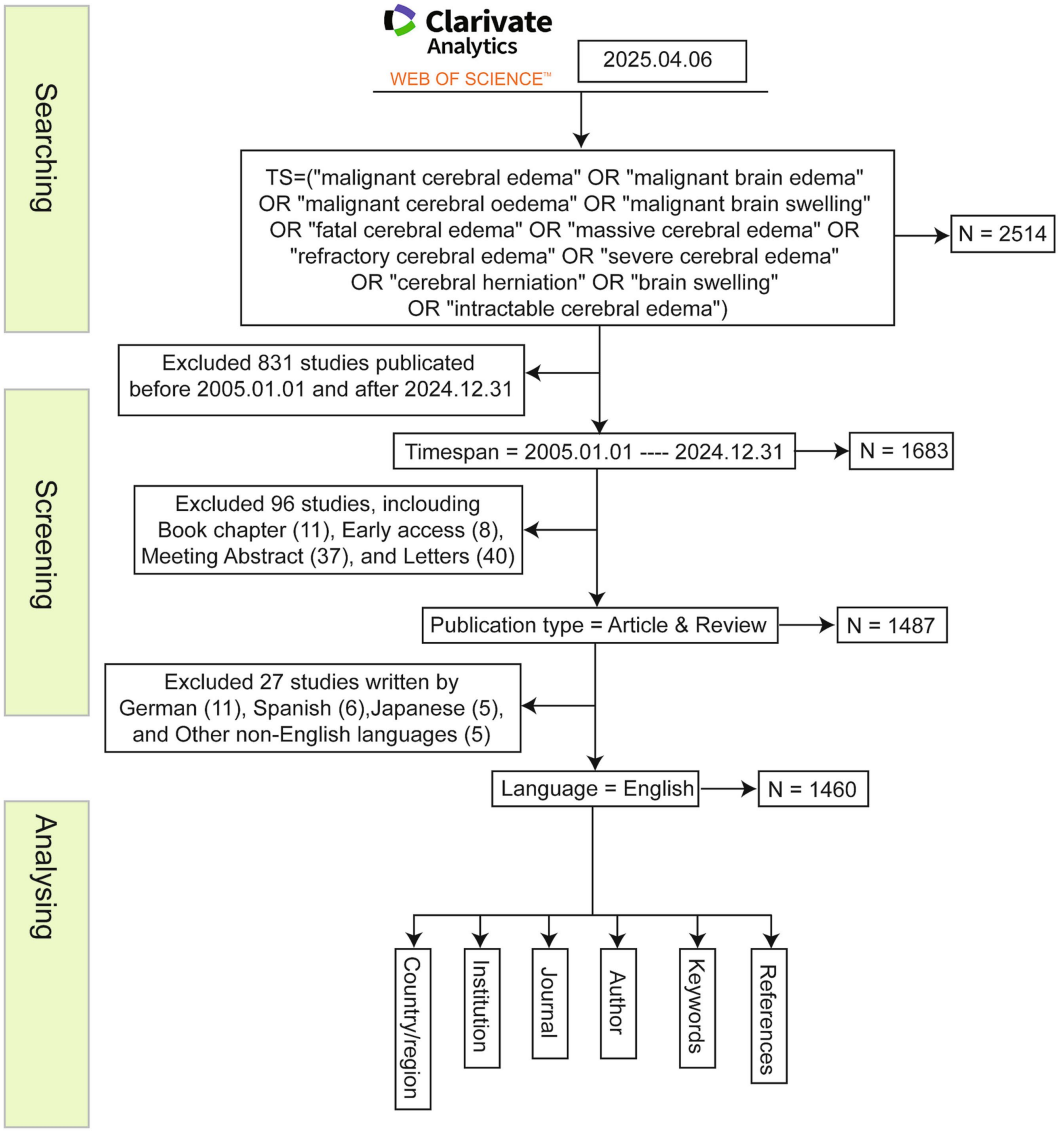
The flowchart of this study.

We systematically retrieved bibliometric records from the WOSCC on April 6, 2025, using a search strategy developed in consultation with a health sciences librarian. The search syntax combined controlled vocabulary (MeSH terms: “Brain Edema”[Mesh] and “Cerebral Herniation”[Mesh]) with free-text keywords identified from a preliminary literature scan: TS = (“malignant cerebral edema” OR “malignant brain edema” OR “malignant cerebral oedema” OR “malignant brain swelling” OR “fatal cerebral edema” OR “massive cerebral edema” OR “refractory cerebral edema” OR “severe cerebral edema” OR “cerebral herniation” OR “brain swelling” OR “intractable cerebral edema”). The initial search yielded 2,514 records. After filtering for publications from January 1, 2005, to December 31, 2024, we retained 1,683 documents. We retained only peer-reviewed articles and systematic reviews published in English to ensure analytical rigor, excluding editorials, conference abstracts, and letters. After deduplication and manual screening, a final dataset of 1,460 records was extracted for analysis (Figure 1). The selection process adhered to Preferred Reporting Items for Systematic Reviews and Meta-analyses Extension for Scoping Reviews guidelines and followed established bibliometric protocols to enhance reproducibility and transparency.

### Data processing and software tools

To ensure cross-platform compatibility, bibliographic data were exported in plain text format, including full metadata and cited references. Data preprocessing was conducted in R software (version 4.4.1) using the Bibliometrix package (16), which standardized author names, institutional affiliations, and keywords. Duplicate entries were removed using DOI and title matching. Three complementary tools were employed for bibliometric analysis: (1) CiteSpace (17) (version 6.4. R1) was used to construct co-citation networks with annual time slicing (2005–2024), using a g-index node selection criterion (*k* = 25) and pathfinder network pruning to identify pivotal publications and emerging trends. (2) VOSviewer (18) (version 1.6.20) generated keyword co-occurrence density maps and author collaboration networks using the LinLog/modularity clustering algorithm (resolution = 1.0, minimum cluster size = 5). (3) The Bibliometrix R package provided performance metrics, including annual publication growth rates, h-index, and international collaboration ratios. It also produced geographic heatmaps and three-field plots to visualize linkages among authors, keywords, and journals. Visualization outputs were optimized through iterative threshold adjustments. For example, keyword maps included only terms occurring ≥ 10 times, and author networks were limited to contributors with ≥ 5 publications.

### Analytical framework

We adopted a multi-dimensional analytical framework to address its objectives. Initially, descriptive statistics were computed to profile annual publication trends, leading journals, and prolific authors or institutions. Second, science mapping techniques were applied to identify foundational research themes and interdisciplinary bridges, including co-citation analysis and keyword co-occurrence clustering. Third, network metrics, including betweenness centrality and modularity scores, were calculated to quantify collaboration patterns and knowledge diffusion pathways. All software configurations and analysis parameters were calibrated based on recommendations from seminal bibliometric methodologies to ensure methodological validity and interpretability of results.

## Results

### Publishing trend analysis

**Figure 2:**
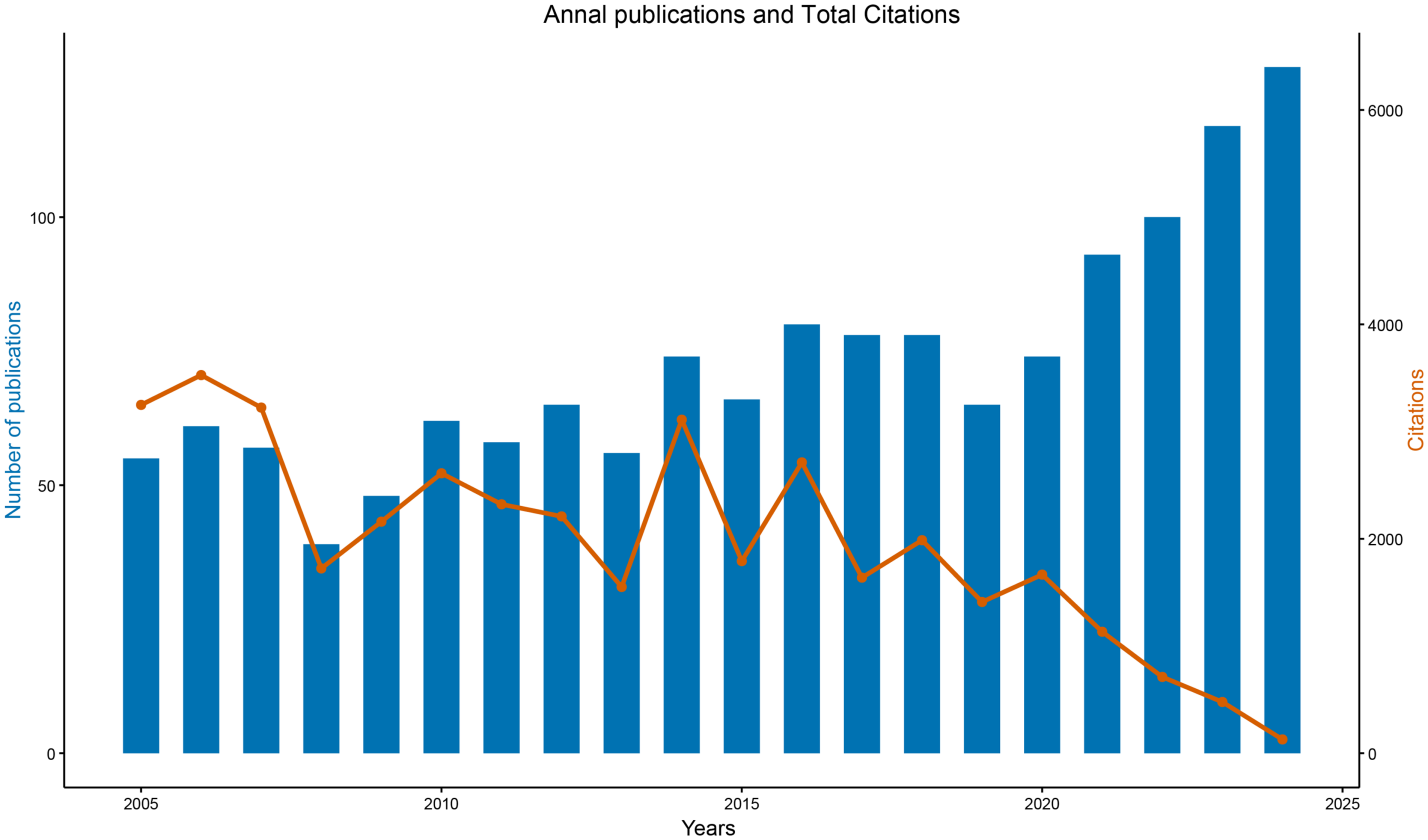
Annual publications and TC in MCE research (2005–2024). Blue bars indicate several articles published each year, while the orange line indicates the TC accrued by papers published in that year.

The bibliographic corpus comprised 1,460 documents published across 560 journals from 2005 to 2024, demonstrating both disciplinary diversity and sustained scholarly engagement. Annual publication output increased from 55 articles in 2005 to 128 in 2024, establishing a consistent upward trajectory. Three distinct growth phases were observed: An initial exploratory phase (2005–2009) with annual publication counts fluctuating between 39 and 57 articles; a consolidation phase (2010–2014) characterized by stabilized outputs (56–74 articles/year); and a rapid expansion phase (2015–2024) during which annual publications exceeded 100 articles from 2022 onward (Figure 2).

Citation analysis identified two prominent peaks—3,527 citations in 2006 and 3,012 citations in 2015—followed by a decline to 129 citations in 2024, consistent with the citation lag commonly observed in recent publications. The field maintained an average citation rate of 26.95 per document, with a median document age of 9 years, underscoring its sustained academic impact (Figure 2). These patterns align with established bibliometric principles governing citation dynamics and disciplinary maturation trajectories.

### Geographical distribution

**Figure 3:**
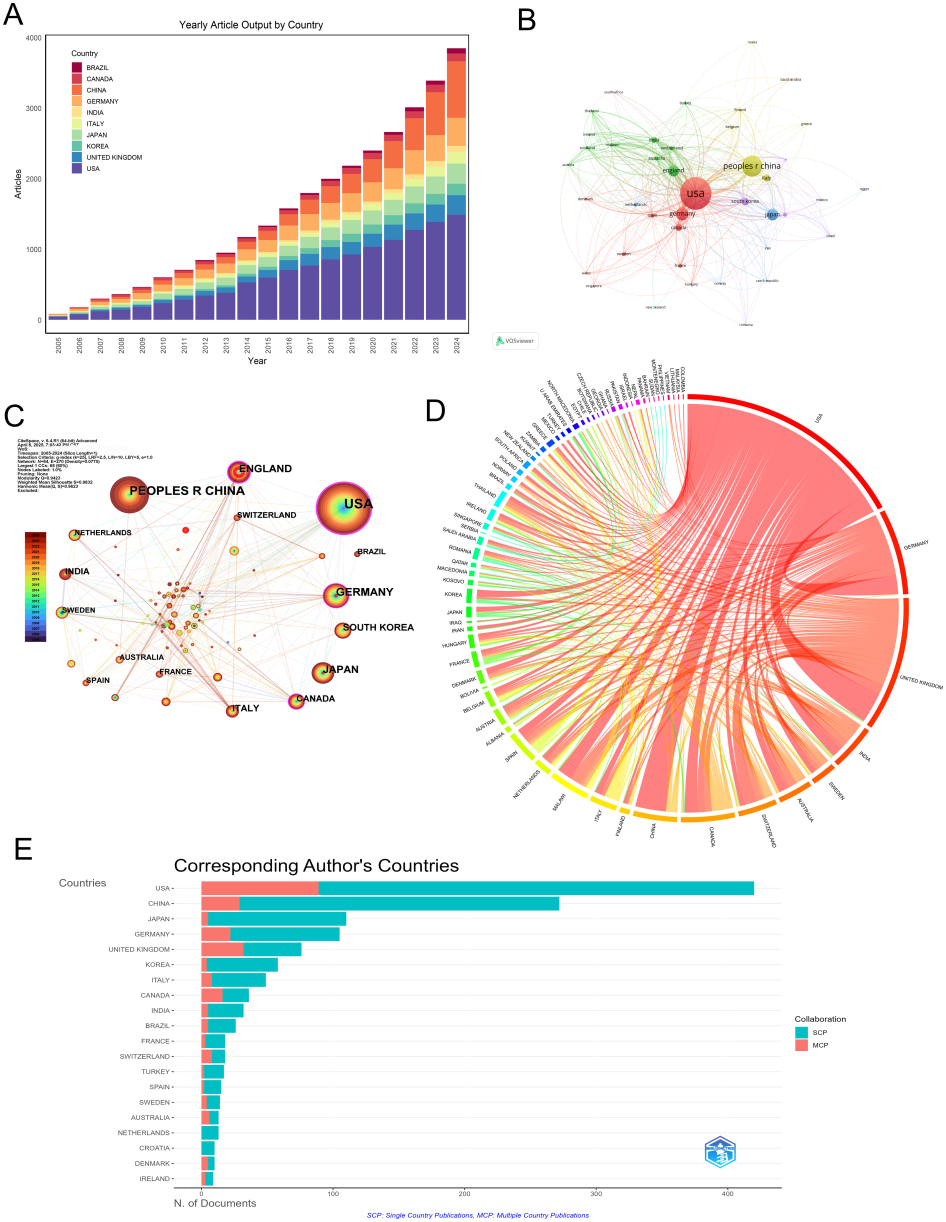
Geographic distribution and international collaboration in MCE research. **(A)** Annual article output (2005–2024) for the top 10 countries. **(B)** Co-authorship network of countries, with node size proportional to publication volume and edge thickness indicating collaboration strength. **(C)** Collaboration density map indicating bilateral partnerships. Node color intensity reflects the mean citation rate of each country, and link width corresponds to the number of joint publications. **(D)** Chord diagram of inter-country collaborations. **(E)** Horizontal bar chart of corresponding author countries, comparing SCP (teal) versus MCP (salmon).

A total of 79 countries contributed to MCE research, with a pronounced concentration in high-income nations. The United States (U.S.) led with 420 publications (28.89%), followed by China (272; 18.71%), Japan (110; 7.57%), and Germany (105; 7.22%) (Figure 3A and Table 1). Longitudinal analysis highlighted sustained output growth in both the U. S. and China, which accounted for 47.6% of global output over the study period.

**Table 1:** Top 10 publication countries.

Country	Articles	Articles %	SCP	MCP	MCP %
USA	420	28.89	331	89	21.19
CHINA	272	18.71	243	29	10.66
JAPAN	110	7.57	105	5	4.55
GERMANY	105	7.22	83	22	20.95
UNITED KINGDOM	76	5.23	44	32	42.11
KOREA	58	3.99	54	4	6.90
ITALY	49	3.37	41	8	16.33
CANADA	36	2.48	20	16	44.44
INDIA	32	2.20	27	5	15.63
BRAZIL	26	1.79	21	5	19.23

Network analyses revealed the USA’s central position, as demonstrated by co-authorship network topology (Figure 3B) and collaboration density mapping (Figure 3C). These visualizations highlight four core nations—the USA, China, Germany, and the UK—exhibiting maximal interconnectivity, forming the structural backbone of global MCE research. Bilateral collaboration patterns were most frequent between the USA and UK (36 publications), followed by the USA-China (35 publications) and USA-Germany (22 publications) partnerships (Figure 3D, Table 2).

**Table 2:** Top 10 country collaborations.

From	To	Frequency
USA	UNITED KINGDOM	36
USA	CHINA	35
USA	GERMANY	22
USA	MALAWI	16
USA	CANADA	15
GERMANY	SWITZERLAND	13
UNITED KINGDOM	MALAWI	12
GERMANY	UNITED KINGDOM	11
USA	INDIA	11
UNITED KINGDOM	INDIA	9

Productivity analysis through the single-country publications (SCP)/multiple-country publications (MCP) framework uncovered divergent collaboration strategies. While the USA maintained leadership in total output (SCP = 331), its international collaboration rate (MCP = 21.19%) was surpassed by Canada (44.44% MCP) and the UK (42.11% MCP), indicating various global research integration (Figure 3E and Table 1).

Citation metrics underscored geographic disparities in scientific influence. The USA dominated both total citations (TC = 16,187) and average citation per article (38.5), followed by the UK (33.1) and Germany (30.5). Denmark exhibited exceptional impact relative to the output size (TC = 683; 68.3 per article). Conversely, China’s citation rate (15.6) remained disproportionate to publication volume, suggesting opportunities for enhanced research visibility (Table 3).

**Table 3:** Citation of top 10 countries.

Country	TC	Average Article Citations
USA	16,187	38.5
CHINA	4,239	15.6
GERMANY	3,203	30.5
UNITED KINGDOM	2,512	33.1
JAPAN	1,632	14.8
KOREA	1,230	21.2
CANADA	1,172	32.6
ITALY	1,096	22.4
FRANCE	853	47.4
DENMARK	683	68.3

### Institutional distribution

A select cohort of academic institutions dominates research productivity in MCE. Harvard University emerged as the leading contributor (139 publications), followed by the University of California System (115 publications) and Harvard Medical School-affiliated institutions (111 publications) (Table 4). A pattern of regional clustering was observed, notably within the University System of Maryland and the University of Maryland, Baltimore, producing 78 and 77 publications, respectively. The Harvard academic ecosystem system further demonstrated its prominence through substantial outputs from Massachusetts General Hospital (73) and Harvard Medical School (61).

**Table 4:** Top 10 publications of institutions.

Affiliation	Articles
HARVARD UNIVERSITY	139
UNIVERSITY OF CALIFORNIA SYSTEM	115
HARVARD UNIVERSITY MEDICAL AFFILIATES	111
UNIVERSITY SYSTEM OF MARYLAND	78
UNIVERSITY OF MARYLAND BALTIMORE	77
MASSACHUSETTS GENERAL HOSPITAL	73
HARVARD MEDICAL SCHOOL	61
UNIVERSITY OF CALIFORNIA SAN FRANCISCO	60
PENNSYLVANIA COMMONWEALTH SYSTEM OF HIGHER EDUCATION (PCSHE)	54
HUMBOLDT UNIVERSITY OF BERLIN	49

**Figure 4:**
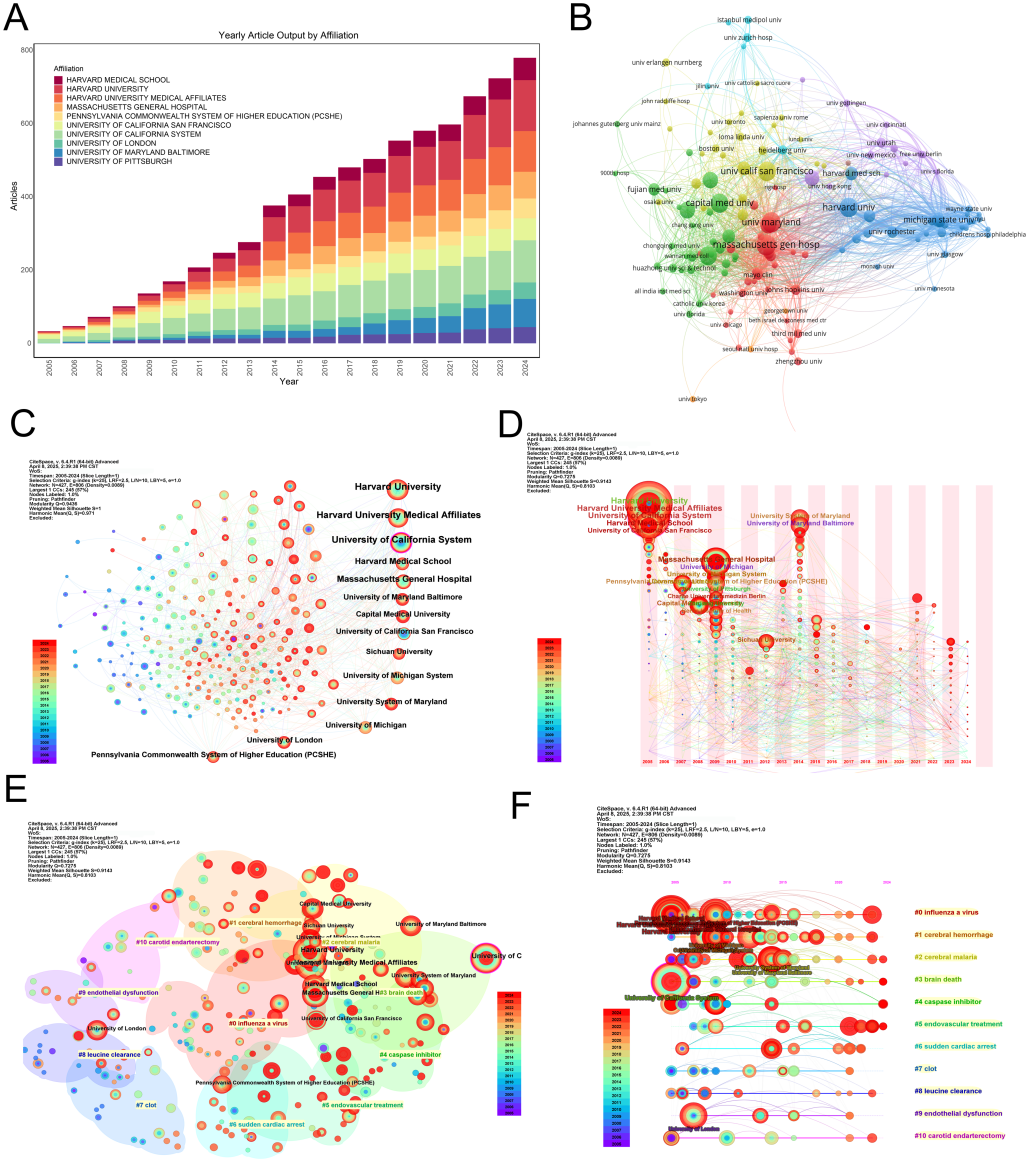
Institutional productivity, collaboration networks, clustering, and thematic evolution in MCE research. **(A)** Stacked annual article output bar chart for the top 10 contributing institutions between 2005 and 2024. **(B)** Institution-level co-authorship network. Node size is proportional to total publications; edge thickness reflects collaboration frequency. Nodes are colored by modularity class, highlighting three major collaborative clusters centered around Harvard (red), the University of California system (blue), and the University of Maryland network (green). **(C)** Overlay visualization of the co-authorship network, with node color indicating the average publication year of each institution and node size proportional to publication volume. **(D)** Bubble-timeline of annual outputs for leading institutions. **(E)** Modularity-based clustering map of institutions. Nodes are sized by total output and colored by the assigned community; semi-transparent halos delineate each cluster. Cluster labels correspond to thematic specializations identified through institutional co-authorship patterns. **(F)** Thematic evolution of institutional research foci over time. Each horizontal track represents one of the 10 major research themes. Bubble size in a given year indicates the volume of publications within that theme, revealing shifts from early molecular studies to emerging clinical and translational topics.

A longitudinal analysis revealed consistent productivity trajectories from top institutions, notably Harvard and the University of California System. Meanwhile, the University of Maryland network has indicated notable growth in publication volume since 2015, potentially reflecting targeted funding initiatives or increased involvement in clinical trials (Figures 4A,D).

Co-authorship network analysis identified Harvard University as the central hub, maintaining extensive collaborative ties with both domestic and international partners (Figure 4B). Secondary nodes—including the University of Maryland and UCSF—functioned as both independent producers and collaborative bridges, particularly when weighted by publication volume (Figure 4C).

Modularity analysis identified three principal institutional clusters influencing MCE research (Figure 4E). The predominant cluster centers on Harvard-affiliated institutions, including Massachusetts General Hospital and Harvard Medical School. These collectively focused on advancing clinical interventions through surgical management optimization and neurocritical care protocol standardization. The second cluster, anchored by the University of California System with significant contributions from UCSF, demonstrated concentrated expertise in translational neuroscience, particularly in elucidating molecular pathways underlying edema formation and developing novel therapeutic agents. The third cluster, led by the University of Maryland network in collaboration with Pennsylvania Commonwealth institutions, focuses on neurocritical care operations research, highlighting population-level outcome studies and evidence-based guideline formulation. These thematic alignments suggest that institutional collaborations are strategically oriented toward complementary research priorities rather than geographical convenience, fostering disciplinary depth and cross-cluster knowledge transfer.

Thematic mapping of institutional outputs revealed distinct research profiles (Figure 4F). Harvard-affiliated centers demonstrated broad expertise across pathophysiology and clinical outcomes, contrasting with the focus of the University of California System on experimental therapies and neuroinflammation. European institutions, including the Humboldt University of Berlin, occupied specialized niches in preclinical modeling, emphasizing geographic variations in research prioritization.

### Journal analysis

**Figure 5:**
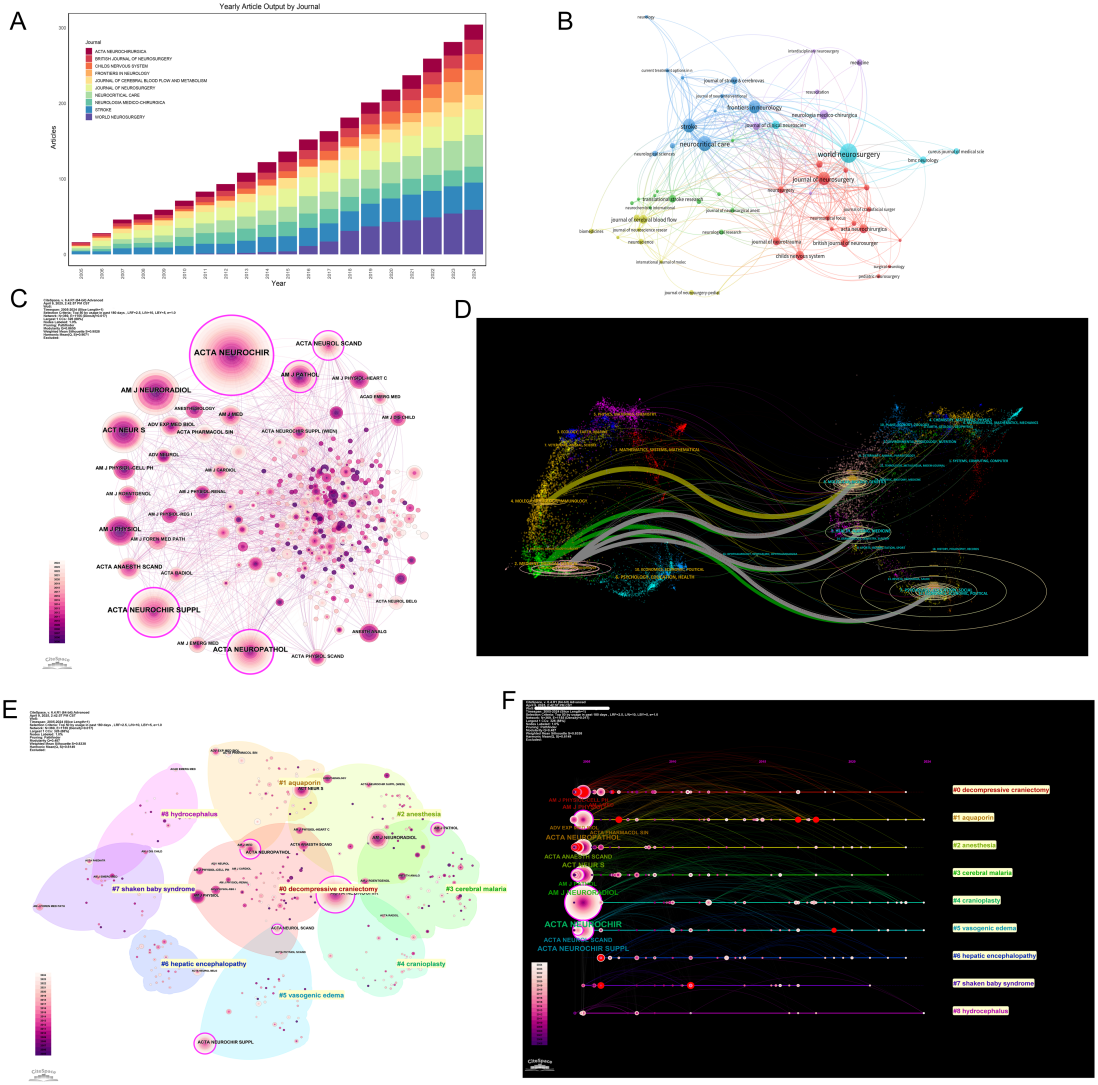
Journal dissemination patterns, co-citation network structure, thematic clustering, and citation bursts in MCE research. **(A)** Annual article output (2005–2024) for the top 10 journals. **(B)** Journal co-citation network. **(C)** Betweenness-centrality overlay on the co-citation network. **(D)** Dual map of MCE research. **(E)** Spatial clustering map of co-cited journals. **(F)** Timeline view of journal citation bursts. Each horizontal track corresponds to a journal experiencing a burst.

The dissemination of MCE research exhibited distinct structural dynamics across academic journals. The leading outlets include World Neurosurgery (59 publications), Neurocritical Care (42 publications), and Stroke (36 publications), accounting for 9.4% of total output (Figure 5A and Table 5). These journals predominantly published clinical studies bridging neurosurgical practice, neurocritical care protocols, and cerebrovascular pathophysiology, reflecting the field’s interdisciplinary foundations.

**Table 5:** Top 10 publications of journals.

Sources	Articles
WORLD NEUROSURGERY	59
NEUROCRITICAL CARE	42
STROKE	36
JOURNAL OF NEUROSURGERY	34
FRONTIERS IN NEUROLOGY	33
CHILDS NERVOUS SYSTEM	21
NEUROLOGIA MEDICO-CHIRURGICA	21
ACTA NEUROCHIRURGICA	20
BRITISH JOURNAL OF NEUROSURGERY	19
JOURNAL OF CEREBRAL BLOOD FLOW AND METABOLISM	19

Co-citation network analysis revealed specialized interconnected journal clusters with thematic orientations (Figure 5B). Stroke and Neurocritical Care were central hubs within citation communities focused on cerebrovascular disease management, while the Journal of Cerebral Blood Flow & Metabolism anchored clusters investigating molecular edema mechanisms. Betweenness centrality metrics confirmed these journals’ pivotal roles in cross-domain knowledge integration (Figure 5C).

Thematic clustering revealed three distinct journal archetypes: (1) Clinical neurosurgery venues (World Neurosurgery, Neurosurgery) emphasizing surgical interventions; (2) neurocritical care platforms (Stroke, Neurocritical Care) specializing in acute management; (3) pathophysiology-focused titles (JCBFM) exploring mechanistic insights (Figure 5E). Keyword alignment patterns further differentiated these clusters. Surgical journals were frequently associated with terms such as “decompressive craniectomy” and “intracranial pressure,” while pathophysiology journals were aligned with keywords such as “blood–brain barrier” and “neuroinflammation” (Figure 5F).

Bibliometric profiling revealed divergent impact trajectories across these publication categories. Despite ranking third in output, Stroke dominated citation metrics (TC = 3,618; h-index = 27), underscoring its role as a high-impact translational nexus. Conversely, World Neurosurgery led publication volume but demonstrated lower citation density (h-index = 17), indicative of its clinical case-report orientation (Table 6). Neurocritical care journals (Neurocritical Care, h-index = 19) balanced productivity with scholarly influence, while surgical titles prioritized dissemination breadth over citation intensity (Table 7).

**Table 6:** The impact of top 10 publication journals, ordered by h-index.

Source	h_index	g_index	m_index	TC	NP	PY_start
STROKE	27	36	1.29	2,618	36	2005
NEUROCRITICAL CARE	20	31	0.95	1,001	42	2005
JOURNAL OF NEUROSURGERY	19	34	0.90	1,669	34	2005
JOURNAL OF CEREBRAL BLOOD FLOW AND METABOLISM	17	19	0.81	1,352	19	2005
WORLD NEUROSURGERY	13	22	0.87	576	59	2011
ACTA NEUROCHIRURGICA	12	20	0.57	537	20	2005
JOURNAL OF NEUROTRAUMA	12	17	0.67	578	17	2008
NEUROSURGERY	11	13	0.52	434	13	2005
PLOS ONE	11	16	0.73	343	16	2011
TRANSLATIONAL STROKE RESEARCH	11	16	0.69	657	16	2010

**Table 7:** Citation of top 10 journals.

Sources	Articles
STROKE	3,618
J NEUROSURG	2,342
NEUROSURGERY	1,408
J CEREBR BLOOD F MET	1,077
NEW ENGL J MED	1,052
J NEUROTRAUM	876
NEUROLOGY	783
ACTA NEUROCHIR	714
LANCET	618
LANCET NEUROL	586

A dual map overlay analysis illustrated cross-domain knowledge flows, revealing predominant citation pathways between molecular biology and clinical medicine domains (Figure 5D). Strong translational feedback loops emerged along clinical-molecular interfaces (green/gray arcs), while limited connectivity existed with computational or physical sciences—a gap suggesting opportunities for enhanced interdisciplinary convergence.

### Author and co-authorship network

**Figure 6:**
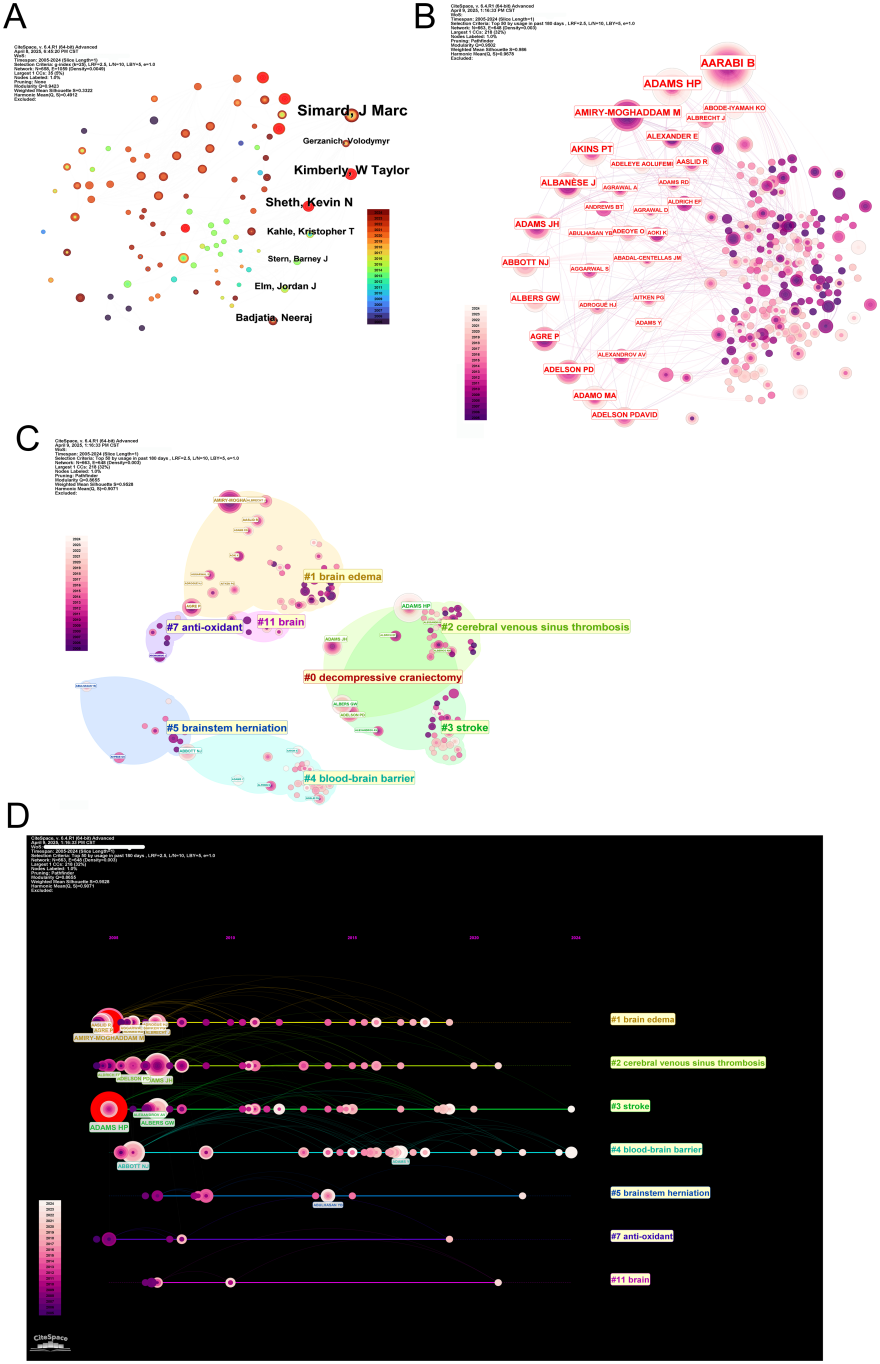
Author productivity, co-authorship networks, clustering, and publication timelines in MCE research. **(A)** Scatterplot of individual authors. **(B)** Co-authorship network overlay highlighting author centrality. **(C)** Modularity-based clustering of the co-authorship network. **(D)** Timeline linkage of top authors to their primary research clusters.

The scholarly landscape of MCE research is shaped by distinct patterns of authorship productivity and influence. Professor SIMARD JM emerges as the most prolific contributor, with 22 publications (NP = 22), maintaining the highest h-index (18) and g-index (9) among active researchers, and a total of 1,032 TC since entering the field in 2010 (Tables 8, 9). Following closely, LIU M (NP = 16), along with HUA Y, KEEP RF, KIMBERLY WT, and VERKMAN AS (NP = 15 each), demonstrate robust productivity. Co-authorship network analysis positions these investigators as central hubs, with SIMARD JM and KIMBERLY WT exhibiting dense collaborative linkages—indicative of leadership within cohesive research consortia (Figure 6A).

**Table 8:** The impact of top 10 publication authors, ordered by h-index.

Author	h_index	g_index	m_index	TC	NP	PY_start
SIMARD JM	18	22	1.13	1,032	22	2010
HUA Y	15	15	0.88	750	15	2009
KEEP RF	15	15	0.88	750	15	2009
VERKMAN AS	15	15	0.71	2,601	15	2005
XI GH	15	15	0.88	750	15	2009
KIMBERLY WT	12	15	1	1,120	15	2014
SHETH KN	12	15	0.8	1,126	15	2011
ALAM HB	10	10	0.71	359	10	2012
TAYLOR TE	10	13	0.83	679	13	2014
GERZANICH V	8	10	0.5	477	10	2010

**Table 9:** Top 10 publication authors.

Authors	Articles
SIMARD JM	22
LIU M	16
HUA Y	15
KEEP RF	15
KIMBERLY WT	15
SHETH KN	15
VERKMAN AS	15
XI GH	15
TAYLOR TE	13
WANG C	13

Notably, scholarly productivity and citation impact follow divergent trajectories. While SHETH KN ranks second in publication volume (NP = 15), their work has accumulated the highest local citation count (131 citations), surpassing KIMBERLY WT (129 citations) and SIMARD JM (96 citations) (Table 10). This discrepancy is mirrored in author co-citation networks, where foundational figures, including AARABI B and SHETH KN, form tightly interconnected citation communities, underscoring their enduring conceptual influence (Figure 6B). Bibliometric profiling further reveals SHETH KN’s strong m-index (0.8), reflecting sustained impact since 2011, despite producing fewer publications than SIMARD JM (m-index = 1.125; Table 8).

**Table 10:** Citation of top 10 authors.

Author	Local Citations
SHETH KN	131
KIMBERLY WT	129
SIMARD JM	96
VERKMAN AS	84
TAYLOR TE	76
LIU M	73
SEYDEL KB	73
WU SM	69
ZHANG SH	66
PAPADOPOULOS MC	65

Thematic mapping of author communities reveals specialized research orientations aligned with keyword clusters (Figure 6C). SIMARD JM’s work predominantly associates with surgical intervention themes (DC cluster), supported by his high h-index (18) and TC (1,032). Contrarily, VERKMAN AS anchors molecular research (BBB cluster), achieving exceptional long-term citation impact (TC = 2,601) despite a lower m-index (0.71), reflecting foundational contributions dating back to 2005. The KIMBERLY WT, with an m-index of 1.0—the highest among mid-career researchers—bridges mechanistic investigations (brain edema cluster) and clinical translation, accruing 1,120 citations since 2014 (Table 8). Spatial proximity between clusters reveals interdisciplinary interfaces, particularly in neuroprotection and cerebral autoregulation research.

Temporal keyword analysis tracks the evolution of scholarly priorities (Figure 6D). The initial dominance of surgical management themes (2000s) gradually gave way to emergent focuses on cerebral venous sinus thrombosis (post-2010) and antioxidant therapies (post-2015). This transition aligns with the entry of investigators, including KIMBERLY WT (PY_start = 2014), whose work exemplifies translational innovation. Meanwhile, veteran researchers, including VERKMAN AS (PY_start = 2005), maintain relevance through sustained mechanistic contributions.

Integrative analysis of productivity patterns, citation networks, and thematic evolution delineates three characteristic author archetypes within MCE research. The first group comprises high-output collaborators exemplified by SIMARD JM (h-index = 18) and the HUA Y/KEEP RF/XI GH research triad (h-index = 15), whose sustained productivity and central network positions anchor large-scale research consortia. The second group comprises conceptual pioneers, including SHETH KN (TC = 1,126) and VERKMAN AS (TC = 2,601), whose seminal contributions continue to shape fundamental disciplinary paradigms. The third group includes translational innovators, including KIMBERLY WT (m-index = 1.0) and GERZANICH V (m-index = 0.5), whose work bridges mechanistic discovery and clinical application, driving thematic shifts toward emerging research frontiers (Table 8). These archetypes emerge not solely from quantitative metrics but through individual scholarly trajectories with evolving disciplinary priorities, ultimately forging a dynamic ecosystem where foundational insights inform clinical translation.

Collectively, these dynamics illustrate how individual scholarly trajectories intersect with broader disciplinary progress, spanning foundational discoveries (VERKMAN AS), clinical implementation (KIMBERLY WT), and collaborative networks (HUA Y, KEEP RF, and XI GH) that amplify collective impact through shared productivity.

### Thematic landscape and evolutionary trajectories

**Figure 7:**
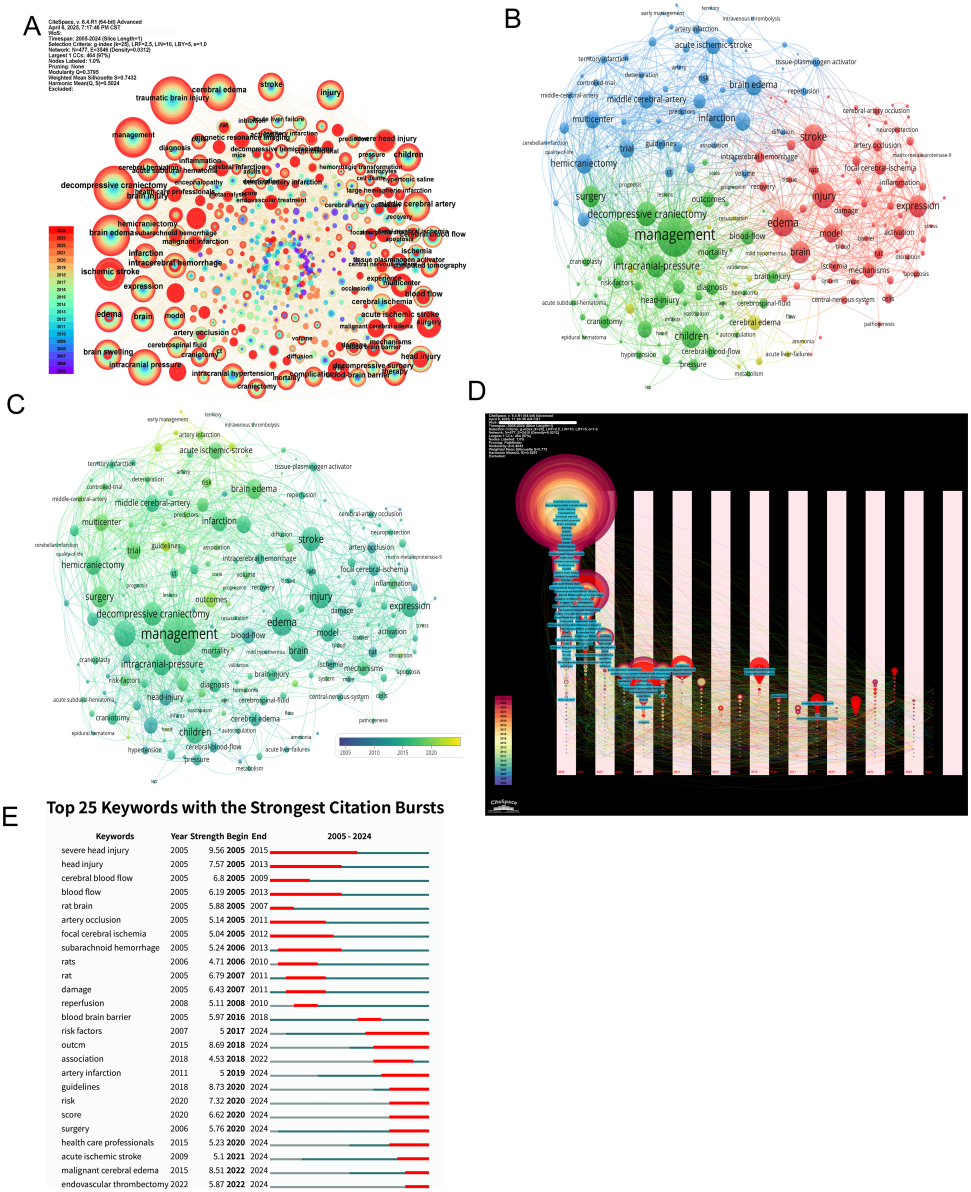
Keyword co-occurrence patterns, thematic clustering, temporal dynamics, and citation bursts in MCE research. **(A)** Global keyword co-occurrence network with node size proportional to co-occurrence frequency. **(B)** Modularity-based clustering of the keyword network. **(C)** Overlay of the co-occurrence network by average occurrence year. **(D)** Temporal evolution map of keyword networks (2005–2024). **(E)** Bar chart of the top 25 keywords with the strongest citation bursts.

Co-occurrence network analysis delineates the conceptual architecture of MCE research, with high-frequency keywords including “management,” “decompressive craniectomy,” and “brain edema” occupying central network positions—highlighting their pivotal role in addressing clinical and mechanistic investigations (Figures 7A–C). Cluster decomposition reveals three dominant thematic orientations: Clinical management strategies focused on surgical interventions, pathophysiological studies exploring molecular mechanisms, and experimental research employing ischemic stroke models (Figure 7B).

Temporal keyword evolution charts a paradigm shift from foundational mechanistic exploration to clinically oriented research priorities. Early-phase studies (2005–2015) emphasized cerebral blood flow dynamics and ischemic injury mechanisms, while recent investigations (2016–2024) increasingly focus on evidence-based guidelines, risk stratification, and healthcare system optimization (Figure 7D). Burst detection analysis corroborates this transition, with persistent citation bursts for “decompressive craniectomy” contrasting against emerging bursts for “healthcare professionals” and “guidelines,” signaling the field’s maturation toward standardized care protocols (Figure 7E).

**Figure 8:**
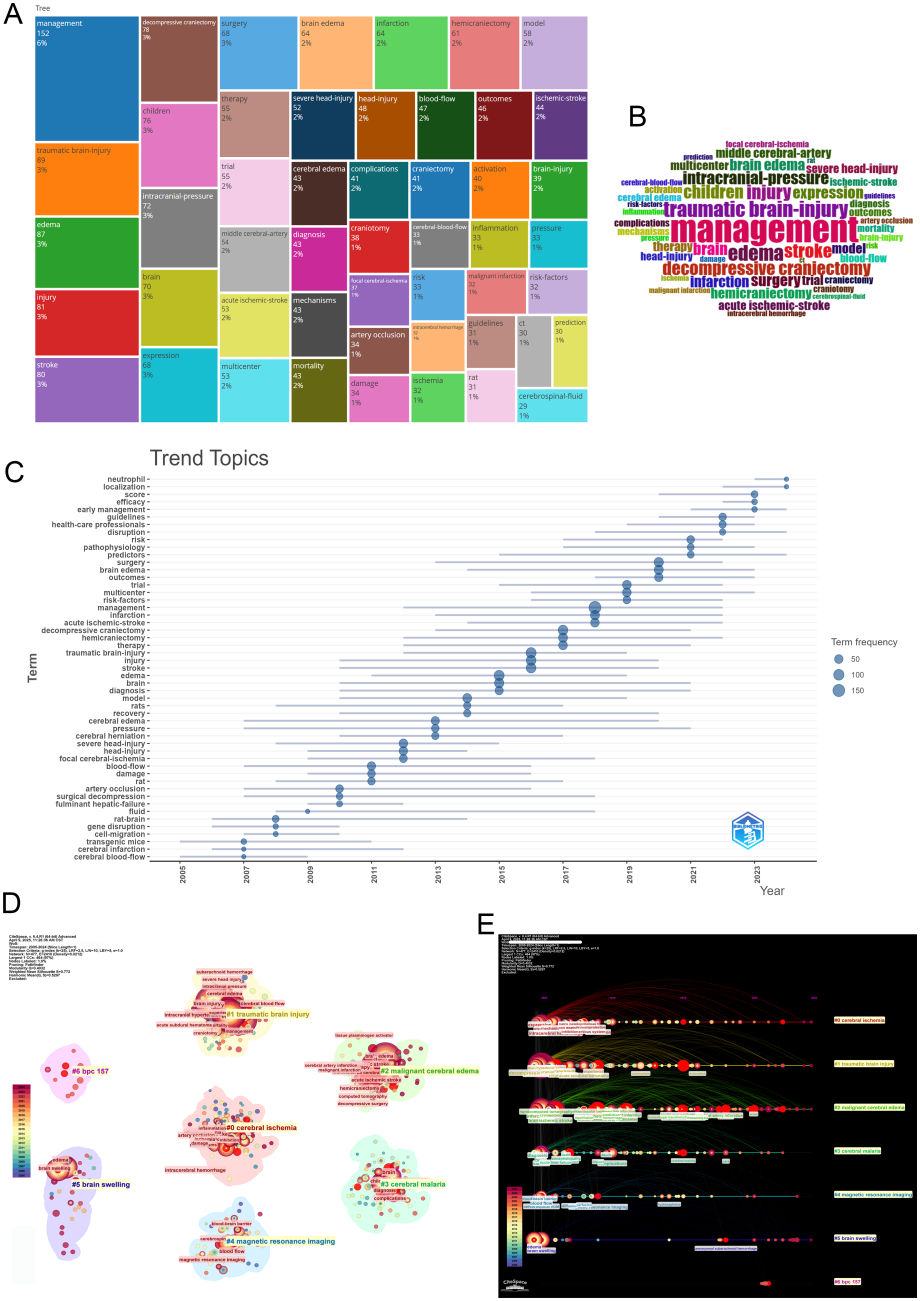
Integrated visualization of keyword prominence, temporal trends, co-occurrence clustering, and co-citation cluster timelines in MCE research. **(A)** Treemap of the most frequent keywords. **(B)** Wordcloud of keywords. **(C)** Trend-topics bubble-plot (2005–2024). **(D)** Keyword co-occurrence network clustering. **(E)** Co-citation cluster timeline view.

Visualization tools further clarify these thematic dynamics. Treemap analysis identifies “management” and “brain edema” as core conceptual anchors, while word cloud projections reinforce their centrality within the research lexicon (Figures 8A,B). Trend topic mapping demonstrates a progressive migration from fundamental vascular pathophysiology toward clinical implementation science, particularly in the ascendance of early intervention strategies and outcome monitoring frameworks over the past decade (Figure 8C).

Clustering analysis highlights the field’s thematic structure through distinct yet interconnected research directions (Figure 8D). Key clusters include managing traumatic brain injury, investigating cerebral ischemia mechanisms, and researching MCE pathophysiology. These clusters exhibit both disciplinary depths—through strong intra-cluster keyword cohesion (“neuroinflammation” and “blood–brain barrier” within the pathophysiology cluster)—and interdisciplinary bridges, particularly between neuroprotective strategies and clinical trial design.

Cluster timeline analysis reveals persistent investigative threads alongside emerging frontiers (Figure 8E). Established domains, including traumatic brain injury management and cerebral ischemia mechanisms, continue to maintain research momentum, while novel themes, including risk factor stratification and neurocritical care operations, have gained momentum post-2015. Thematic interfaces emerge at cluster peripheries, particularly between neuroinflammatory pathways and clinical decision-making algorithms, suggesting fertile ground for translational innovation.

Collectively, these analytical dimensions trace the field’s evolution from pathophysiological discovery to therapeutic application—a trajectory marked by an increasing emphasis on guideline-driven management, multidisciplinary care models, and patient-centered outcomes. This maturation pattern mirrors broader trends in neurocritical care, where mechanistic insights increasingly inform precision medicine approaches across the translational continuum.

### Citation and co-citation analysis

**Figure 9:**
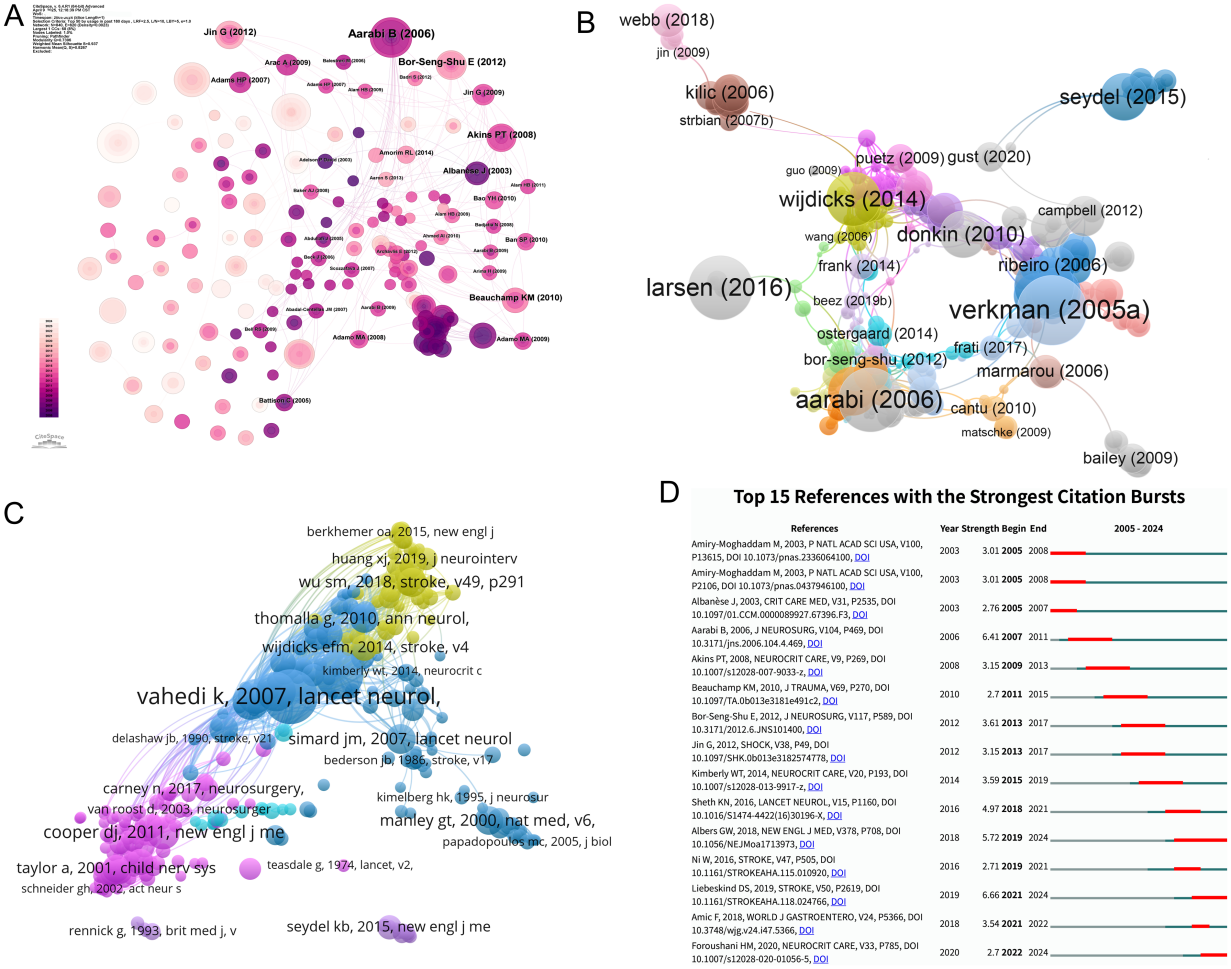
Reference co-citation network structure, clustering, temporal overlay, and strongest citation bursts in MCE research. **(A)** Global co-citation network of references. **(B)** Modularity-based clustering of the co-citation network. **(C)** Temporal overlay of the co-citation network. **(D)** Bar chart of the top 15 references with the strongest citation bursts.

The citation landscape of MCE research is shaped by pivotal studies that have influenced clinical practice and mechanistic understanding. WU SM et al.’s 2018 systematic review (45 citations) established early prediction models as critical tools for guiding therapeutic interventions following post-stroke MCE (12), while AARABI B et al.’s 2006 prognostic evaluation of DC in traumatic brain injury (38 citations) remains foundational for surgical decision-making (19). Concurrently, WIJDICKS EFM et al.’s 2014 evidence-based guidelines (38 citations) standardized neurocritical care protocols, reflecting the field’s maturation toward consensus-driven management (20). Notably, four of the ten most-cited articles originated from Stroke, underscoring the journal’s central role in MCE scholarship (Figures 9A,B and Table 11). This citation trajectory mirrors the discipline’s evolution from exploratory pathophysiology to precision medicine frameworks integrating risk stratification and artificial intelligence (AI)-enhanced predictive modeling.

**Table 11:** Citation of top 10 references.

**Rank**	**Author**	**Title**	**Year**	**Citation counts**	**Journal**
1	WU SM et al.	Early Prediction of Malignant Brain Edema After Ischemic Stroke: A Systematic Review and Meta-Analysis	2018	45	Stroke
2	AARABI B et al.	Outcome following decompressive craniectomy for malignant swelling due to severe head injury	2006	38	Journal of Neurosurgery
3	WIJDICKS EFM et al.	Recommendations for the Management of Cerebral and Cerebellar Infarction with Swelling: A Statement for Healthcare Professionals from the American Heart Association/American Stroke Association	2014	38	Stroke
4	SEYDEL KB et al.	Brain Swelling and Death in Children with Cerebral Malaria	2015	34	The New England Journal of Medicine
5	HUANG XJ et al.	Predictors of malignant brain edema after mechanical thrombectomy for acute ischemic stroke	2019	29	Journal of Neurointerventional Surgery
6	STIVER SI et al.	Complications of decompressive craniectomy for traumatic brain injury	2009	27	Neurosurgical focus
7	JO K et al.	A simple prediction score system for malignant brain edema progression in large hemispheric infarction	2017	23	PloS one
8	BATTEY TWK et al.	Brain edema predicts outcome after nonlacunar ischemic stroke	2014	21	Stroke
9	PAPADOPOULOS MC et al.	Aquaporin-4 gene disruption in mice reduces brain swelling and mortality in pneumococcal meningitis	2005	17	The Journal of Biological Chemistry
10	SIMARD JM et al.	Glibenclamide is superior to decompressive craniectomy in a rat model of malignant stroke	2010	17	Stroke

Co-citation analysis reveals enduring intellectual foundations, with Stroke and The Lancet Neurology dominating among high-impact references. The seminal pooled analysis by VAHEDI K et al. (2007; 116 co-citations) validated early decompressive hemicraniectomy for malignant middle cerebral artery infarction by synthesizing three randomized controlled trials, setting a methodological benchmark for subsequent clinical trials (4). Complementary studies by HACKE W et al.’s 1996 prognostic marker study (94 co-citations) (21) and HOFMEIJER J et al.’s HAMLET trial (2009; 71 co-citations) (9) collectively established decompressive surgery as a life-saving intervention while quantifying functional outcome benefits. These studies, which predominantly address malignant infarction and traumatic edema, highlight the field’s emphasis on multicenter randomized designs to achieve higher evidence levels (Figure 9C and Table 12).

**Table 12:** Co-citation of top 10 references.

**Rank**	**Author**	**Title**	**Year**	**Citation counts**	**Journal**
1	VAHEDI K et al.	Early decompressive surgery in malignant infarction of the middle cerebral artery: a pooled analysis of three randomized controlled trials	2007	116	The Lancet. Neurology
2	HACKE W et al.	‘Malignant’ middle cerebral artery territory infarction: clinical course and prognostic signs	1996	94	Archives of Neurology
3	HOFMEIJER J et al.	Surgical decompression for space-occupying cerebral infarction (the Hemicraniectomy After Middle Cerebral Artery infarction with Life-threatening Edema Trial [HAMLET]): a multicenter, open, randomized trial	2009	71	The Lancet. Neurology
4	JUTTLER E et al.	Decompressive Surgery for the Treatment of Malignant Infarction of the Middle Cerebral Artery (DESTINY): a randomized, controlled trial	2007	63	Stroke
5	VAHEDI K et al.	Sequential-design, multicenter, randomized, controlled trial of early decompressive craniectomy in malignant middle cerebral artery infarction (DECIMAL Trial)	2007	58	Stroke
6	COOPER DJ et al.	Decompressive craniectomy in diffuse traumatic brain injury	2011	53	The New England Journal of Medicine
7	SIMARD JM et al.	Brain edema in focal ischemia: molecular pathophysiology and theoretical implications	2007	46	The Lancet. Neurology
8	WU SM et al.	Early Prediction of Malignant Brain Edema After Ischemic Stroke	2018	45	Stroke
9	MANLEY GT et al.	Aquaporin-4 deletion in mice reduces brain edema after acute water intoxication and ischemic stroke	2000	43	Nature Medicine
10	SCHWAB S et al.	Early hemicraniectomy in patients with complete middle cerebral artery infarction	1998	43	Stroke

Temporal burst analysis delineates shifting intellectual priorities (Figure 9D). Early mechanistic studies, including those by Amiry–Moghaddam (2003), dominated 2005–2008 citations (burst strength = 3.01) (22, 23), whereas Liebeskind (2019) exhibited the strongest recent burst (6.66; 2021–2024), signaling a growing interest in AI-driven prediction models (3). The sustained influence of Albers (2018) (24) and Ni (2016) (25) post-2016 correlated with therapeutic innovation cycles, particularly in neuroprotective agent development and multimodal monitoring integration. This citation chronology traces the field’s pathophysiological exploration to translational implementation, where computational analytics increasingly inform personalized management paradigms.

## Discussion

### Insights into publication dynamics and field maturation

This bibliometric analysis systematically examined the evolution, structure, and impact of research within MCE over the past two decades. Analyzing 1,460 publications across 560 journals from 2005 to 2024 reveals a clear growth trajectory, diversification, and scholarly engagement trajectory. The findings highlight a transition from early mechanistic investigations to more clinically oriented studies characterized by increasing international collaboration and institutional specialization. The U.S. emerged as the dominant contributor in both productivity and citation impact, while institutions, including Harvard University and the University of California System, formed the backbone of global research networks. Thematic clustering and keyword analysis further identified shifts in research priorities—from surgical interventions toward evidence-based management and translational innovation. These insights collectively illustrate the maturation of MCE research and underscore the growing importance of interdisciplinary collaboration in advancing therapeutic strategies.

### Critical analysis of global research inequities

High-income countries, particularly the U.S., China, Japan, and Germany, dominate the field, collectively contributing approximately two-thirds of all publications. This concentration reflects disparities in research infrastructure, funding availability, and clinical trial capacity. The U.S., accounting for 28.89% of total output, also occupies a central position in international collaboration networks, underscoring its role as both a knowledge producer and a global connector. However, collaboration strategies vary significantly: while the U.S. leads in total output, nations including Canada and the UK demonstrate higher rates of international co-authorship, suggesting a reliance on transnational networks to amplify visibility and resource access. Citation analysis further illustrates uneven scientific influence. The U.S., Germany, and the UK maintain high citation densities, indicative of impactful contributions, whereas China exhibits a relatively low citation rate despite its high volume—highlighting potential gaps in research visibility, originality, or dissemination strategies. Interestingly, smaller countries, including Denmark, achieve exceptional per-article impact, reflecting focused, high-quality research efforts. These geographic trends underscore the importance of promoting equitable research collaboration, capacity-building in emerging regions, and strategic dissemination efforts to enhance global knowledge integration in MCE research.

### Role of academic powerhouses in shaping research trajectories

Harvard University, its affiliated institutions, and the University of California and the University System of Maryland account for a substantial proportion of research output, reflecting historical academic leadership and strategic investment in neurocritical care research. These institutions serve as prolific contributors and are central nodes in collaborative networks, driving knowledge dissemination and innovation through synergistic partnerships. Modularity analysis revealed three thematically distinct institutional clusters—centered around Harvard, the University of California, and the Maryland system—aligning with specific research priorities, including surgical intervention optimization, translational neurobiology, and population-level outcomes research. This specialization underscores shifting from geographically driven collaboration toward goal-oriented, complementary research integration. Moreover, emerging institutions, including the University of Maryland network, have demonstrated accelerated growth in recent years, likely supported by increased clinical trial involvement and targeted funding mechanisms. These patterns highlight both the benefits of concentrated expertise and the potential for the broader inclusion of rising research centers in shaping the future landscape of MCE science.

### Dissemination patterns and knowledge transfer mechanisms

A small group of high-output journals—World Neurosurgery, Neurocritical Care, and Stroke—serve as central dissemination platforms, collectively reflecting the clinical and translational breadth of the field. Thematic clustering of these journals indicates a clear division of labor: Surgical journals focus on procedural and case-based content, neurocritical care outlets emphasize acute clinical management, while pathophysiology journals explore molecular mechanisms. Despite high publication volume, journals, including World Neurosurgery, exhibit lower citation densities, highlighting a potential trade-off between clinical accessibility and scholarly impact. Contrarily, Stroke demonstrates moderate output and excellent citation metrics, underscoring its role as a translational nexus between clinical practice and mechanistic research. Dual map overlay analysis reveals robust citation flows between molecular biology and clinical medicine yet limited integration with computational or engineering sciences— highlighting missed opportunities for cross-disciplinary innovation. These patterns reflect the strengths and limitations of current dissemination pathways and underscore the requirement for more inclusive journal ecosystems that address experimental, clinical, and technological domains to fully leverage the multi-dimensional nature of MCE research.

### Scholarly leadership and collaborative synergies

A small cohort of prolific investigators—led by SIMARD JM, the most productive author with the highest h-index and g-index—anchors the collaborative backbone of the field through sustained output and network centrality. However, citation patterns reveal that productivity does not always equate to influence. Researchers, including SHETH KN and VERKMAN AS, exemplify conceptual leadership, having shaped foundational paradigms through highly cited, domain-defining contributions despite lower publication volumes. Thematic mapping and co-authorship analyses explain three archetypes of scholarly engagement: High-output collaborators, including SIMARD JM and the HUA Y/KEEP RF triad, who drive consortium-based productivity; foundational theorists, including VERKMAN AS, whose early mechanistic insights continue to resonate; and translational innovators, including KIMBERLY WT, whose entry into the field coincides with shifts toward emerging therapeutic directions. These distinctions reflect not only individual academic trajectories but also broader structural forces guiding the evolution of MCE research—from surgical management to molecular and systems-level interventions. The temporal evolution of author-keyword alignments further demonstrates this transition, highlighting the role of mid-career leaders in fostering interdisciplinary convergence. Collectively, these authorial dynamics suggest that sustained field advancement hinges on synergistic interactions between legacy expertise, translational vision, and collaborative capacity.

### Conceptual mapping of evolving research priorities

Early investigations focused on foundational pathophysiologies—including cerebral blood flow dynamics and ischemic injury—but have gradually shifted toward studies emphasizing guideline development, healthcare delivery optimization, and patient-centered care. Keyword co-occurrence and burst analyses underscore this transition, with an enduring focus on “decompressive craniectomy,” now complemented by emerging emphases on “guidelines” and “healthcare professionals.” This thematic shift reflects a broader disciplinary maturation, where mechanistic insights are increasingly translated into standardized clinical protocols. Clustering and trend mapping analyses highlight three interconnected research pillars: surgical management, molecular pathogenesis, and preclinical modeling. These domains are anchored by core conceptual terms—“management” and “brain edema”—that unify the field’s lexicon while supporting disciplinary differentiation. Notably, recent thematic interfaces between neuroinflammatory pathways and operational decision-making frameworks signal the rise of translational frontiers driven by interdisciplinary convergence and data-informed care strategies. Ultimately, these trajectories portray a research landscape that not only diversifies in scope but also deepens in clinical relevance, mirroring the neurocritical care field’s broader shift toward precision, integration, and outcome-driven innovation.

### Foundational works and emerging intellectual Frontiers

Highly cited studies—including WU SM et al.’s review on predictive modeling, AARABI B et al.’s prognostic assessment of DC, and WIJDICKS EFM et al.’s neurocritical care guidelines—illustrate the field’s maturation toward standardized, evidence-based care. Co-citation analysis highlights a core group of landmark studies, most notably VAHEDI K’s pooled analysis and the HAMLET trial, which collectively established surgical decompression as a life-saving intervention supported by randomized clinical evidence. The centrality of journals, including Stroke and The Lancet Neurology, within co-citation networks reinforces their status as key conduits for high-impact scholarship. Temporal burst analysis illustrates a shift in research priorities—from early mechanistic studies to current emphases on AI-enhanced prediction, therapeutic innovation, and personalized monitoring. The growing influence of recent contributors, including Liebeskind and Albers, reflects the field’s convergence with computational analytics and multimodal decision-making. These citation trajectories reflect the discipline’s ongoing evolution, increasingly defined by integrative, data-informed approaches to individualized patient care.

### Research hotspot

#### Molecular mechanisms and translation

Recent pharmacologic research on cerebral edema has zeroed in on agents that modulate the molecular pathways of fluid accumulation, but clinical efficacy remains inconsistent (26). SUR1-TRPM4 channel inhibitors (27), aquaporin antagonists, glucocorticoids, and tranexamic acid can shrink vasogenic edema in animal models and small human studies, yet larger trials have shown only modest volume reduction without durable functional gains. Outside the brain, retina-directed therapies such as 4D-150 gene therapy for wet age-related macular degeneration (wAMD) and UBX1325 for diabetic macular edema have posted encouraging early results, hinting at broader applications for edema control (28, 29).

Mechanistically, attention has shifted from a purely BBB perspective to a more integrated view that includes neuroinflammatory cascades and cell-type–specific responses (30). Up-regulation of AQP4 water channels, for example, tracks with edema severity after stroke and TBI, but blocking AQP4 in humans has not reproduced the benefits seen in rodents—underscoring how cytotoxic and vasogenic edema differ across diagnoses and species (31). Likewise, anti-inflammatory strategies help in some models but appear secondary in others, suggesting that inflammation is a context-dependent rather than universal therapeutic target (32).

Bridging these gaps will require finer patient stratification and better translational tools. Single-cell sequencing and brain-organoid models can map glia-neuronal networks that drive fluid shifts, while BBB-penetrant small molecules may deliver targeted inhibition more effectively (33). Future trials should incorporate these biologic insights, apply precision enrollment criteria, and test combination regimens that align therapy with the specific edema subtype and disease stage.

#### Translational challenges

The middle cerebral artery occlusion (MCAO) model remains central to MCE research, as it closely replicates the pathological features of large territorial infarctions in humans, including progressive cerebral edema and neurological deficits. This model has been widely used to evaluate the efficacy of therapeutic agents, including mannitol and hypertonic saline (34). Recent MCAO model studies have indicated that NKCC1 inhibitors, including bumetanide, can alleviate cerebral edema by modulating ionic homeostasis (35). However, clinical failures—including that of the free radical scavenger NXY-059—have underscored the limitations of animal models, which often fail to replicate the heterogeneity and comorbidities of human brain tissue (36). To address this challenge, it is crucial to develop more sophisticated *in vitro* systems, including three-dimensional BBB chips, and to integrate multi-omics platforms—combining transcriptomics and metabolomics—to enhance the predictive power of drug screening (37, 38). Moreover, combinational therapies targeting neuroinflammation and oxidative stress represent a promising strategy to overcome the limitations of current single-target treatments (39).

#### Surgical intervention optimization

Current clinical research increasingly focuses on the precision application of DC in patients with malignant middle cerebral artery infarction. Several randomized controlled trials, including HAMLET and DESTINY, have demonstrated that DC can reduce mortality rates from approximately 80–30% (4, 9). The timing of surgery (ideally within 48 h of stroke onset) and standardized indications—including infarct volume and degree of midline shift—is critical for optimizing patient selection (40). However, the long-term functional benefits remain debated. Some studies suggest that the procedure significantly improves mortality, while others question its efficacy in improving long-term neurological outcomes, particularly in older patients or those with poor preoperative neurological status (41, 42). This ongoing debate underscores the need for more refined patient selection criteria and further investigation into the timing and long-term outcomes of surgical interventions in brain edema management.

While DC rapidly lowers intracranial pressure and improves cerebral perfusion (43), it is associated with postoperative complications, including cerebrospinal fluid leakage and infections, and a lack of a unified framework for functional outcome assessment. Moving forward, the development of multimodal decision-making models—integrating biomarkers including S100B and GFAP with automated imaging analysis—could enhance patient stratification and predict surgical benefit more accurately (44). Additionally, combining DC with therapeutic hypothermia or neuroprotective agents may represent a promising strategy to optimize clinical outcomes (45, 46).

#### Clinical pathway balancing

Current guidelines, including those from the Neurocritical Care Society, recommend a tiered intervention strategy for managing cerebral edema: First-line use of hyperosmolar agents (mannitol or hypertonic saline), second-line decompressive surgery, and third-line adjunctive hypothermia therapy (47). Standardized protocols have significantly reduced treatment delays (average reduction of 2.1 h from symptom recognition to intervention) (48). However, institutional preferences for hyperosmolar agents remain divided, given concerns about mannitol-associated nephrotoxicity and hypertonic saline-induced electrolyte imbalances (49). Risk-stratified individualized protocols—including the EDEMA score, which integrates NIHSS score, infarct volume, and blood glucose levels—are proposed to identify high-risk patients (50). Future efforts should focus on optimizing guidelines using real-world data and developing AI-driven clinical decision support systems to balance standardization with patient-specific needs (51). Additionally, tailored therapeutic approaches for special populations (pregnant individuals, immunocompromised patients) require urgent refinement (52).

#### AI in predictive modeling

AI-driven imaging and predictive modeling are rapidly transforming cerebral edema care: automated biomarkers derived from routine CT scans correlate more strongly with standard edema endpoints than manual measures, while deep-learning analysis of hyperattenuated imaging markers (HIM) improves early identification of malignant edema (53–55). Building on these advances, multimodal machine-learning models—using long short-term memory networks or random forests to fuse imaging features (midline shift, ADC), physiologic signals (intracranial pressure, blood-pressure variability), and laboratory indices (lactate, D-dimer)—already achieve an AUC of about 0.92 for ultra-early (<6 h) prediction of malignant cerebral edema and even distinguish inflammation-dominant from metabolic-dysfunction subtypes (56). Yet clinical adoption lags because most models remain “black boxes” trained on single-center datasets (57); future work must embrace federated learning for cross-institutional data sharing, embed explainable-AI tools such as SHAP values or attention maps to reveal key predictors, and integrate real-time decision support into ICU monitoring systems to build clinician trust and enable next-generation (41), precision edema management.

### Limitations

Our study has several limitations, consistent with the inherent constraints of bibliometric methodologies. First, the exclusive reliance on WOSCC and English-language publications may have omitted regionally significant studies published in non-English journals or non-indexed databases, potentially skewing geographic productivity trends. Second, inconsistencies in author and institutional name formats within WOSCC (variations in abbreviations or affiliations) could lead to fragmented representation of research contributions. Finally, while our search strategy combined controlled vocabulary and free-text terms, absolute precision in topic relevance screening cannot be guaranteed, as some publications may address MCE only peripherally rather than as a primary focus. Nevertheless, our rigorous methodology and large-scale dataset provide a firm overview of the field’s evolution, capturing dominant trends and collaborative networks with high fidelity.

## Conclusion

This bibliometric analysis synthesizes two decades of MCE research, revealing a field transitioning from foundational pathophysiological insights to clinically actionable innovations. While advancements in DC protocols and AI-driven predictive models have improved survival outcomes, critical gaps persist—particularly in pediatric research, biomarker discovery, and equitable global collaboration. The dominance of high-income countries in research output contrasts starkly with the disproportionate burden of MCE-related mortality in low-resource settings, underscoring systemic disparities in knowledge production and dissemination. Moreover, limited integration with computational sciences highlights the untapped potential for AI-enhanced frameworks in risk stratification and personalized care. Future efforts must prioritize translational validation of preclinical targets, establishing equitable partnerships to bridge geographic divides, and ethical implementation of AI to transform survival gains into functional recovery globally. By addressing these imperatives, the MCE research community can advance toward precision medicine paradigms that ensure equitable benefits across diverse populations.

## Data Availability

The raw data supporting the conclusions of this article will be made available by the authors, without undue reservation.
